# P65 Optimizing antibiotic prescribing in enteric fever: a systematic review of clinical, laboratory, and microbiological factors influencing treatment outcomes

**DOI:** 10.1093/jacamr/dlag102.071

**Published:** 2026-06-26

**Authors:** Fatima Saqib, Rasha Abdelsalam Elshenawy

**Affiliations:** School of Health, Medicine and Life Sciences, University of Hertfordshire, College Lane, Hatfield AL10 9AB, UK; School of Health, Medicine and Life Sciences, University of Hertfordshire, College Lane, Hatfield AL10 9AB, UK

## Abstract

**Background:**

MDR and XDR *Salmonella enterica* have severely limited empirical antibiotic options in enteric fever, particularly in low- and middle-income countries (LMICs) where the disease remains endemic [1]. Under the WHO AWaRe classification, Access-class antibiotics are no longer reliably effective, forcing clinicians to escalate to Watch- and Reserve-class agents with greater resistance potential [2]. To effectively implement antimicrobial stewardship, there is an urgent need to understand the clinical variables that affect antibiotic prescribing decisions, to optimize prescribing and reduce the emergence of antimicrobial resistance in clinical settings globally.

**Objectives:**

To identify clinical, laboratory, and microbiological variables associated with enteric fever diagnosis and severity; to evaluate their influence on empirical antibiotic selection and treatment outcomes; and to compare reported antibiotic regimens against the WHO AWaRe classification.

**Methods:**

A systematic review was conducted following PRISMA 2020 guidelines across Medline via OVID, Scopus, and Cochrane Library (February 2026). The PICO framework guided inclusion: patients with confirmed or suspected typhoidal Salmonella infection, empirical antibiotic interventions, comparisons across AWaRe classes, and outcomes of treatment success, failure, or relapse. Studies addressing non-typhoidal Salmonella or vaccine-only strategies were excluded. Data were extracted into a standardized spreadsheet. No ethical approval was required as all data were sourced from published literature.

**Results:**

From 326 screened studies, 14 met inclusion criteria (*n*≈3319 patients), comprising 8 RCTs (57%), 3 observational studies, 1 individual patient data meta-analysis, 1 controlled human infection study, and 1 case series. Eleven studies (79%) were conducted in LMICs, predominantly Nepal (*n*=4), India (*n*=3), and Pakistan (*n*=3), and three in high-income countries (UK, USA). Paediatric populations were the most frequently studied group (6 studies, 43%). Regarding the WHO AWaRe classification, Access-class agents appeared only as resistance comparators, confirming their loss of monotherapy utility. Watch-class antibiotics dominated prescribing throughout 2016–2025, principally ceftriaxone (10 studies) and azithromycin (9 studies). Reserve-class carbapenems did not appear until 2023, coinciding with XDR S. Typhi emergence. Fluoroquinolone resistance was the strongest predictor of treatment failure (80% versus 0%, *P*<0.0001), with prolonged fever clearance (8.2 versus 3.8 days). Blood culture positivity significantly predicted outcome; culture-positive patients had higher failure rates with gatifloxacin than ceftriaxone (26% versus 7%, *P*=0.01). Oral azithromycin achieved equivalent cure rates to IV ceftriaxone (95.2% versus 93.7%) while reducing hospital stay by 2.1 days (*P*=0.001). In Pakistan, XDR prevalence reached 65.7%, making Reserve-class carbapenems unavoidable, with fever duration of 8–14 days and age under 10 years as key escalation triggers.

**Conclusions:**

This study establishes that fluoroquinolone resistance status, blood culture positivity, XDR confirmation, patient age, fever duration, and travel history are critical determinants of empirical antibiotic selection in enteric fever. The progressive loss of Access-class efficacy and the unavoidable escalation to Reserve-class carbapenems in XDR-endemic settings represent a serious and escalating threat to global AWaRe stewardship goals. These findings support development of a structured, AWaRe-aligned prescribing framework to effectively implement antimicrobial stewardship, standardize empirical decision-making, save patient lives, and reduce antimicrobial resistance globally.
